# Having Their Cake and Eating it Too: An Exploratory Mixed-Methods Study on the Dietary Behaviours of Family Carers of Persons With Dementia

**DOI:** 10.1177/14713012251355832

**Published:** 2025-07-23

**Authors:** Michael Smith, Natalie Dickinson, Nicholas Sculthorpe, Louise Ritchie, Rachel Kimble

**Affiliations:** 1 Sport and Physical Activity Research Institute, 6413 The University of the West of Scotland, Blantyre, UK; 2 Alzheimer Scotland Centre for Policy and Practice, 6413 The University of the West of Scotland, Blantyre, UK; 3 Adult Nursing, Community and Health, 6413 The University of the West of Scotland, Paisley, UK

**Keywords:** carer, dementia, nutrition, mediterranean diet, diet quality, health

## Abstract

**Introduction:** While dementia caregiving is known to place considerable demands on carers, its impact on carers’ own dietary behaviours and nutritional health remains poorly understood. Understanding how caregiving impacts carers’ diet quality is essential to inform supportive interventions. Purpose: This study aimed to explore the diet quality of family carers of persons with dementia and identify caregiving-related factors influencing their nutrition. **Methods:** A concurrent mixed-methods design was employed with 30 family carers completing a quantitative online survey and 10 participating in qualitative semi-structured interviews to provide contextual depth. The online survey included demographic data, dietary intake via a validated 24-h recall, diet quality using the 14-Item Mediterranean Diet Adherence Screener (MEDAS), and food insecurity using the Food Insecurity Experience Scale (FIES). **Results:** Family carers were food secure according to the FIES, and their overall diet quality was moderate, with a mean MEDAS score of 6.6 ± 2.1. Eighty percent exceeded saturated fat and 43% exceeded free sugar intake recommendations. Only 33% of carers reported eating ≥5 portions of fruit and vegetables, with 23% meeting intake of dietary fibre. The findings from the semi-structured interviews demonstrated that caring for a family member with dementia could influence their own dietary behaviours, due to the practical aspects of caregiving and the evolving palates and capabilities of the person with dementia. **Conclusion:** Family carers of persons with dementia often fail to meet dietary recommendations despite moderate diet quality, with emotional burden, time scarcity, and competing priorities significantly influencing their own eating behaviours. These findings highlight the need for flexible, personalised interventions, such as digital tools, meal delivery services, embedded nutrition support, and peer network, that address carers’ emotional stress, time constraints, and caregiving roles to improve their dietary behaviours.

## Introduction

Dementia is a complex, debilitating brain disorder that causes a progressive decline in memory, communication, cognition, and eventually, the ability to carry out simple everyday tasks (Sehar et al., 2022). Approximately 900,000 people in the UK are estimated to have dementia, the prevalence of which is due to increase exponentially as the population ages (Wittenberg et al., 2019). With the increasing prevalence of dementia, the number of carers needed to support persons with dementia in their daily activities is also on the rise. Informal (unpaid) carers, predominantly family members, form the backbone of dementia care, providing essential support valued at approximately £13.9 billion annually in the UK (Wittenberg et al., 2019). Despite their critical role in caregiving, the health and well-being of family carers often go unaddressed. It is well-documented that family carers have poorer mental and physical health than non-carers (Richardson et al., 2013), highlighting the need for targeted strategies to address these health disparities.

A healthy diet or better diet quality, defined as a higher intake of fruits, vegetables, fish, and whole grains, is widely recognised as a cornerstone of good physical and mental health, reducing the risk of chronic diseases and supporting overall well-being (Afshin et al., 2019; Guasch-Ferré & Willett, 2021; Jaron & Galal, 2009; Laraia, 2013; Owen & Corfe, 2017; Slawson et al., 2013). However, research focussing on the dietary behaviours of carers of persons with dementia remains limited, despite evidence suggesting that carers are particularly vulnerable to malnutrition and suboptimal dietary patterns. Previous studies demonstrate that carers exhibit ‘at risk’ dietary behaviours such as increased reliance on coffee for energy (Tsapanou et al., 2024), inadequate intake of fruit and vegetables (Bailey et al., 2019) and susceptibility to both undernutrition (Rullier et al., 2014; Tombini et al., 2016), and over-nutrition, for example, obesity (Carpenter et al., 2020; Vitaliano et al., 1996). Nevertheless, to our knowledge, no studies have examined the quality of carers’ diets, which may offer more meaningful insights into multidimensional dietary behaviours, which are more informative for health outcomes than isolated measures of certain nutrients (Hu, 2002).

Moreover, while previous studies suggest that family carers are at risk of poor dietary health, there is a lack of contextual understanding of how caregiving stressors and constraints contribute to these behaviours. For example, family carers of persons with dementia face unique challenges that may influence diet quality, including the physical and emotional demands of caregiving, financial pressures, and changes in meal preparation routines due to the altered eating behaviours and appetites of the person with dementia (Cipriani et al., 2016; Kasper et al., 2015; Walker-Clarke et al., 2022). Caregiving often requires carers to prioritise the nutritional needs of their loved ones over their own, leading to compromised dietary behaviours (Uemura & Hirakawa, 2020). In a recent study, Mole et al. (2021) explored family carers’ experiences of providing nutritional care for persons with dementia and noted that carers reported sacrificing their own nutrition to meet the needs of the person they cared for. However, to what extent this impacts the carers’ overall diet quality remains underexplored. In this context, the primary aim of this study was to pilot a mixed-methods approach to provide a more comprehensive understanding of the dietary behaviours of family carers of persons with dementia.

## Methods

### Study Design

A convergent conceptual framework was developed to address the research question ‘What is the impact of caring for a person with dementia on dietary quality of informal carers?’ The framework was developed around three constructs:

(1) Quantitative assessment of diet quality, measured using validated tools.
(2) Qualitative exploration of how caring for a person with dementia influences dietary behaviours.
(3) Integration of datasets to identify patterns or divergences within the sample population.

Pragmatism is the philosophical perspective most commonly associated with mixed-methods research designs (Creswell & Clark, 2017) and this worldview was adopted by the authors, acknowledging the problem-based nature of the research goal. This study utilised a concurrent mixed-methods design using an online survey to capture quantitative data on dietary intake and food security, and semi-structured interviews to capture qualitative data on the influence of caring on dietary behaviours in a subset of survey respondents.

### Participants and Recruitment

Participants voluntarily took part in the study that was advertised on social media and shared through dementia and caring organisations. Inclusion criteria were UK-based primary, informal carers (≥18 years of age), responsible for providing daily nutritional care to a community-dwelling family member (≥65 years of age), with a confirmed diagnosis of dementia. Participants were not required to reside with the care recipient to avoid limiting the recruitment pool and ensuring that a variety of familial relationships could be explored. Exclusion criteria were carers with medical dietary restrictions or requirements. As this was an exploratory study to gather preliminary insights, 30 carers were recruited for the online survey which is in-line with the recommendations due to the pilot nature of this study (Johanson & Brooks, 2010), 10 of whom were then invited to participate in an interview. A purposive sampling method for the interviews was used with the intention of obtaining a balance in terms of carer sex, relationships, and living arrangements. Informed consent was obtained from all participants before their involvement in the survey and interview; the study was approved by the Health and Life Sciences Academic Integrity and Ethics Committee (#21672).

### Quantitative Component

A self-completed online survey was distributed using QuestionPro (©2024) from March – September 2024. The survey collected participant demographic information such as age, self-reported BMI, postcode, employment status, education, relationship to the person with dementia, hours of support provided per day, length of caring for the person with dementia and the person with dementia's age at diagnosis. The survey included validated questionnaires on diet quality and food insecurity. Participants then completed an online dietary assessment tool accessed via an embedded link in the survey and entering personalised login credentials.

#### Diet Quality

The Mediterranean Diet Adherence Screener (MEDAS) is a widely used tool for evaluating overall diet quality by assessing adherence to the Mediterranean diet which has been associated with reduced disease risk (Miller et al., 2020). It consists of 14 items that measure key components of the diet, such as the intake of fruits, vegetables, olive oil, fish, legumes, nuts, and wine, while also considering the moderation of certain foods like red meat, sugary baked goods or drinks, and dairy products and preference of certain foods such as olive oil as a main source of fat and vegetable protein or white meat over red meat. Each item is scored as 0 or 1, with a maximum score of 14, where higher scores reflect better adherence and higher diet quality; with low adherence, ≤5; moderate to fair adherence, 6–9; good or very good adherence ≥10 (Martínez-González et al., 2012).

#### Food Insecurity

The Food Insecurity Experience Scale (FIES), developed by the United Nations Food and Agriculture Organization (UN FAO), provides a globally comparable measure of the severity of food insecurity. It comprises eight questions that assess respondents’ self-reported challenges in accessing adequate food. Based on the number of affirmative responses, individuals are classified into one of four categories: food secure, mildly food insecure, moderately food insecure, or severely food insecure. These classifications represent distinct points along the continuum of food security that can be compared to the UK population (Pool & Dooris, 2022).

#### Dietary Intake

Intake24, a validated (Foster et al., 2019), self-completed 24-h recall system that allows users to report all foods and beverages consumed in the previous 24 hours was used to capture participants’ dietary intake. It prompts participants to provide detailed information on portion sizes and preparation methods, using visual aids for accuracy. Foods within the system are linked to the Nutrient Databank and all data are automatically coded so that nutrient intake can be estimated. Intake24 is currently being piloted in the Scottish Health Survey and is the dietary assessment method for the National Diet and Nutrition Survey (NDNS) Rolling Programme.

### Qualitative Component

Semi-structured, one-to-one interviews were conducted to capture information related to the influence of caring on dietary behaviours (n = 10) using a moderator guide developed by the research team referencing previous research interested in dietary behaviours of carers (Lindeza et al., 2024; Rullier et al., 2014; Uemura & Hirakawa, 2020). Ten interviews were thought likely to be sufficient to reach data saturation, particularly given the purposive nature of participant selection The interview explored the carers’ knowledge and perceptions of a healthy diet, perceived and actual barriers to healthy eating; and finally, factors that might facilitate and encourage healthy eating as a response to caring for a person with dementia (Supplemental File 1). The interviews were conducted online over Microsoft Teams due to participants’ diverse regional distribution (Saarijärvi & Bratt, 2021). Interviews were transcribed real-time using the in-built function on Microsoft Teams and then checked for accuracy against the audio recordings.

### Data Analysis

Quantitative data was analysed using Jamovi (Version 2.4.8). Descriptive statistics are reported as means (± standard deviations) for continuous data unless otherwise stated. For categorical data, percentages and frequencies were calculated.

Qualitative interview responses were analysed using reflexive thematic analysis, engaging in researcher reflexivity to acknowledge biases, and collaboratively identifying patterns that captured the lived experiences and dietary behaviours of family carers of persons with dementia. (Braun et al., 2023). The data analysis was conducted iteratively and followed the six stages of reflexive thematic analysis: (a) familiarisation; (b) coding; (c) generating initial themes; (d) developing and reviewing themes; (e) refining, defining, and naming themes; and (f) writing up (Braun & Clarke, 2006) facilitated using NVivo 12 software (QSR International). MS familiarised himself with the data and led the coding and generation of initial themes. Ongoing iterative discussions were held within the research team to develop and refine the named themes, thereby reducing potential researcher bias.

Quantitative and qualitative findings were then integrated in the analysis through side-by-side examination of the data sets as a whole, to facilitate understanding of macro-level comparisons and contrasts between the data sets (Creswell & Clark, 2017).

## Results

### Survey Findings

#### Participant Demographics

Thirty carers aged 43−80 years completed the survey. The survey participants’ characteristics are presented in Table 1. Seventy-seven percent of the respondents were female, 53% were an adult child of the person with dementia and 53% were not living with the person with dementia. Self-reported BMI was optionally entered if participants knew their current height and weight (n = 25). Sixteen (64%) family carers’ BMI was classified as overweight or obese. All respondents were classified as being food secure as per the FIES.

**Table 1.** Characteristics of Survey Respondents (*n* = 30)

Sex	
Female	23 (77%)
Male	7 (23%)
Age (yrs)	63 ± 9
BMI (kg/m^2^)^a^	25.6 ± 3.9
Index of multiple deprivation; median (IQR)	7 (4)
Highest level of education	
High school or equivalent	12 (40%)
Bachelor’s degree	10 (33%)
Postgraduate degree	8 (27%)
Employment status	
Retired	13 (43%)
Full-time	8 (27%)
Part-time	6 (20%)
Unemployed	2 (7%)
Other	1 (3%)
Relationship with person with dementia	
Adult child	16 (53%)
Spouse	12 (40%)
Partner	1 (3%)
Daughter-in-law	1 (3%)
Carer lives with person with dementia	
Yes	14 (47%)
No	16 (53%)
Time spent caring for person with dementia per day (hrs)^b^; median (IQR)	4 (14)
Length of time as carer for person with dementia (months); median (IQR)	33 (36)
Person with dementia’s age at diagnosis (yrs); median (IQR)	75 (15.5)
Food insecurity experience scale	
Food secure	30 (100%)

Data are mean ± SD or n (%) unless otherwise stated.

^a^*n* = 25 due to missing response data.

^b^*n* = 29 due to missing response data.

#### Diet Quality

**Figure 1.**
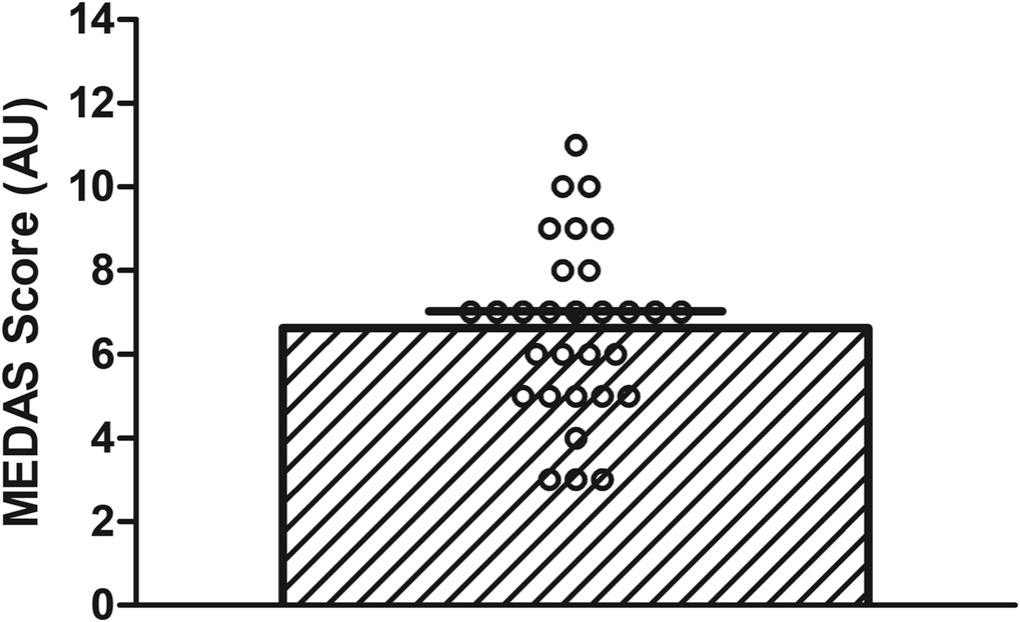
Individual, Mean, and Standard Deviation of Mediterranean Diet Adherence Screener (MEDAS) Questionnaire Score (Arbitrary Units) of Carers (n = 30). The Maximum Possible Score is 14 if all Components are Achieved

**Figure 2.**
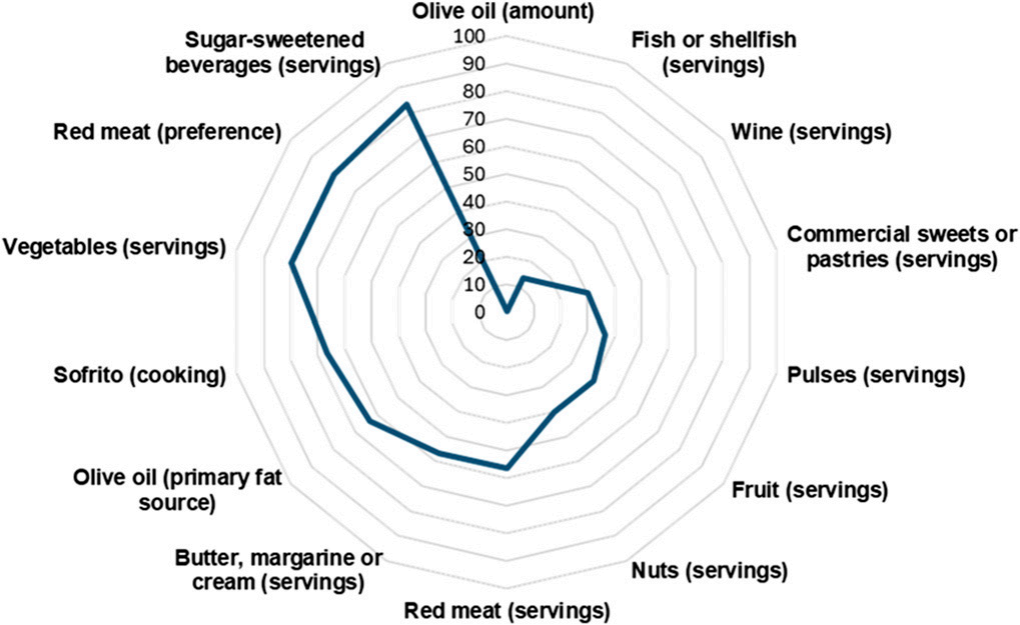
Radar Chart of the Percentage of Carers (n = 30) Who Scored for Each of the 14 Mediterranean Diet Adherence Screener (MEDAS) Items. A Score of One Indicates Healthy Dietary Patterns in Line With the Mediterranean Diet

The mean MEDAS score was 6.6 ± 2.06 (range 3−11) arbitrary units, as shown in Figure 1. Thirty percent of family carers reported low adherence, 60% fair adherence and 10% high adherence to the Mediterranean dietary pattern. A radar chart is shown in Figure 2 to visualise which MEDAS components were least/most likely to be achieved. Scores were plotted as a percentage of carers who scored points for each of the individual components.

#### Dietary Intake

The dietary intake of the carers of persons with dementia is shown in Table 2. Carers’ energy (kcal/day) and fibre (g/day) intake and average daily portions of fruit and vegetables were below current recommendations. Only 33% of carers reported eating ≥5 portions of fruit and vegetables and 23% ≥ 30 g of fibre. Conversely, the proportion of saturated fat as a percentage of energy intake and g/day of free sugars exceeded the guidelines; by 80% and 43% of carers, respectively.

**Table 2.** Dietary Intake Based on 24-H Dietary Recall Using Intake24

	Carers intake	Recommendations based on current UK guidelines
Energy intake (kcal)	Males: 2192 ± 380 Females: 1595 ± 730	Males: 2294-2342 kcal Females: 1840-1912 kcal
Carbohydrate (% of EI)	44.7 ± 10.6	∼50% of EI
Free sugars (% of EI)	7.5 ± 7.5	<5% of EI
AOAC fiber intake (g)	20.3 ± 9.7	30 g
Protein (g)	Males: 76.2 ± 19.8 Females: 73.0 ± 38.3	Males: 53.3 g Females: 46.5 g
Fat (% of EI)	35.6 ± 11.2	∼35% of EI
Saturated fat (% of EI)	12.2 ± 4.4	Maximum 10% of EI
Fruit and vegetables (portions)	4.8 ± 3.7	≥5
Nuts (g)	6.3 ± 12.1	N/A
Olive oil (g)	0 ± 0	N/A

Data are mean ± SD. EI; energy intake.1 portion of fruit or vegetables estimated at 80g. Recommendations based on SACN statement on nutrition and older adults living in the community (SACN, 2021) given the age demographic of the survey respondents.

### Qualitative Findings

Ten carers (6 female and 4 male) who completed the survey participated in one-to-one interviews which lasted between 32 and 64 minutes.

The analysis of the interview data created two main themes, with subthemes. The first theme, ‘A lot on our plates’ represents how the day-to-day and practical aspects of caregiving impact on the diets of family carers. The second theme ‘Shifting p(a)lates’ represents the specific impact caring for someone with dementia has on diet and nutrition overall.

### Theme 1 - A Lot on Our Plates

It is well established that providing care for a person with an illness or a disability can impact the physical and mental well-being of the person providing care. Participants discussed the impact of caregiving on their own diets. This included how they changed their eating patterns to meet the needs of the person they were caring for, the emotional and physical impact of caregiving, and how dealing with their own health problems further exacerbates their diets and nutrition.

#### Changing Routines and Food Availability

Participants recognised that providing care for a family member with dementia impacted on the food choices they made, as well as their eating patterns. Tiredness due to balancing caring with other responsibilities, as well as a desire to ensure that the nutritional needs of the person with dementia were met, resulted in their own preferences and routines being overlooked as they attempted to fit in with the likes and routines of the person they cared for. This was particularly prominent in situations where the carer lived in the same household as the person with dementia.

But rather than be bothered with cooking two meals I would make the spaghetti bolognese and I’d have some as well. (Husband, Age 69, live-in carer)

This often resulted in participants reporting less flexibility in their eating habits, as well as routine eating, which removes some of the enjoyment and social aspect of eating rituals.

So, one of one of my observations, I think this is true of most people with dementia, but certainly true of my wife, is that regularity is quite key. So, I’d say that one of the things that’s happened because of my caring role, is that we’re eating meals at a more sort of regular time. So, we’re having breakfast at the same time, lunch at the same time, evening meal at round about the same time. Whereas in the past, I think particularly with the evening meal, there might have been a bigger flexibility. (Husband, Age 69, live-in carer)

Dietary behaviours are influenced by commensality and participation in social groups, where shared rituals around food become part of the social experience. Historical or current attitudes to nutrition and food appeared to be a good predictor of how well the carer was able to maintain a healthy lifestyle despite the other potential factors from their caring role that could influence their diet.

I’m very mindful of what we eat. I am. I am, I am. Have done … watched my weight for donkey’s years. (Wife, Age 62, live-in carer)

When family carers lived in a different household from the person living with dementia, there was a focus on ensuring the person with dementia was eating but trying to avoid eating themselves to prevent them from overeating or making poorer dietary choices whilst balancing two households.

It would be awful to live with her and have to... Oh, God. No. Oh, yeah. Oh, dear me. Yeah. It would be awful. Yeah. Trying to balance my own diet and health needs with hers. It just can't. They just. God knows how I would manage that. (Daughter, Age 54, Live-out carer)

Another commonly cited approach was family carers missing meals all together.

I might miss food or not eat so much. Perhaps, like yesterday I went around early in the morning. I ended up not eating many breakfast wise, but that’s as likely to be because I'm busy at work than anything else, I suppose. (Daughter, Age 58, Live-out carer)

However, some participants acknowledged the benefits of routine eating to support them to maintain the energy required for caregiving.

A healthy diet for me would be eating regularly. Because when I’m tired, I will just have one pile of crap in the evening and eat nothing in between. So regular eating is the key for me. (Daughter, Age 54, Live-out carer)

Changes in routine and flexibility around eating for the person with dementia have indirect impacts on the diets of carers, by removing some of the spontaneity around food for them. This is particularly true for those who live with their care partner, who have to adapt to encourage regular mealtimes for their family member.

#### Emotional Eating (Disordered Eating)

Many of the participants spoke about the difficulties of caring for a person living with dementia. High levels of stress and the emotional toll of caring for a loved one could lead carers to using eating or alcohol consumption as a coping mechanism.

I’m frustrated or, you know, if, if, if [person with dementia] is having a confused moment and it’s very stressful you know, to, to join her in having some ice cream or, you know, to have a few glasses of wine is, you know, I would, I would go that way. Whereas if I didn’t have that, you know, those stressor moments. Then I might not have done that. (Husband, Age 69, Live-in carer)

This can result in carers recognising their poor nutritional choices and feeling guilty about comfort eating.

You’d be pissed off and resentful that you’ve spent your whole day doing something. She appreciates it. She can’t help it. But you know what I mean. So, I would just think, stuff it. I’m having [chocolate bar]. I’m going to buy five donuts from the [supermarket] on the way home. Donuts are a bad habit as well. There’s a theme. And just eat that, and it makes you feel better for about 1.5 seconds after you’ve finished, and then you just feel disgusting. (Daughter, Age 54, Live-out carer)

The emotional impact of caring can lead to disordered eating or drinking habits, including comfort eating, binge eating and consumption of alcohol.

#### Family Carers’ General Health and Morbidities

The general health of carers, as well as their own morbidities impacted on their ability to manage their diets and nutrition as well as their ability to provide care. They were very aware of trying to maintain their own health to allow them to continue to provide care for their loved one, particularly when they were a spouse or partner of the person with dementia, and cognizant of the impact of age-related illnesses.

I think the main thing to do is to eat what you enjoy and try not to get overweight. Because you still want to keep your mobility and try to avoid any of the obvious things from being overweight, just getting arthritis or diabetes, if you can avoid it. (Husband, Age 80, Live-in carer).

This is often challenging, and increased tiredness and lower mood can impact the motivation to maintain a healthy diet.

If you have a poorer diet, it affects how you’re feeling. It can affect your mood; you can become low. You can put on weight. You can become lethargic, loss of energy. Patience, I imagine, would change. You have to be on top of the game to think ahead to what he’s going to do all the time. I’m up three or four times in a night because he’s off wandering. (Wife, Age 62, Live-in carer)

Further, feelings of helplessness around health, as participants aged, were evident. Some participants stated that maintaining a healthy diet would no longer benefit them, and the priority was to use food as sustenance to have enough energy to get through the day.

The idea of eating healthily at this stage in my life is ridiculous. I mean, focussing on a healthy diet at our stage of life becomes something which is totally unnecessary. Food becomes much less of, how do I say it, it's a less important part of the day, it’s something you’ve just got to get out of the way. (Husband, Age 80, Live-in carer).

However, there were instances where the physical toll of caregiving was impacting the health of the family carer and exacerbating underlying health problems.

I’ve got post-concussion headaches and stuff like that. I keep forgetting things, and post-traumatic stress and… post-traumatic stress and fibromyalgia, chronic fatigue. But it’s difficult to decide which bit is down to the dementia caring and which bit is down to my condition. (Husband, Age 58, Live-in carer).

Overall, the health, situation and impact of caring for a loved one with dementia impacts the diets and nutrition of family carers. While there are some positive impacts, family carers reported having so much ‘on their plates’ due to caring, and prioritising the needs of their family member often meant that the carers’ health, diet and nutrition were not prioritised.

### Theme 2 - Shifting P(a)lates

As well as the impact of general caregiving on the diets and nutrition of participants, they also reported specific impacts related to how dementia affects the loved one they are caring for.

#### Evolving Palates

A common symptom of dementia is a change in response to certain tastes and textures of food. This means that foods or drinks that a person living with dementia enjoyed pre-diagnosis or earlier in their diagnosis become dislikes and can cause stress and distress responses when served. Most often this means moving towards a preference for ‘bland’ and ‘sweet’ foods. Some carers reported compromising their diets or omitting foods to accommodate their family members’ evolving palates or abilities to eat certain foods.

“I used to eat a lot more fish, for example, and I’m eating much less fish than I used to eat. My wife doesn't. She doesn’t like the texture of fish. And especially oily fish. Now, I used to really enjoy mackerel and sardines and things like that, and huge kippers, kippers are one of my favourite things, and she can't stand them. So, there’s quite a few things we just don’t buy anymore, which we would previously have bought. So, I end up omitting certain things.” (Husband, Age 80, Live-in carer).

Additionally, the shift to more sweet foods resulted in increased availability of things like chocolate, cakes, and sweets in the household, and often social outings would centre around going to get some cake.

I mean, we never really ate sweets or chocolate much as a family. But he’s desperate to have chocolate. He must have chocolate every day. And, you know, I don’t eat much chocolate. But you know, I buy chocolate for him all the time. (Female partner, Age 75, Live-out carer).

This not only increases the temptation for family carers, but also a need to join the person with dementia eating cake to connect socially, when they would not have previously.

So we go for a drive, and then we always end up getting cake. So, whether I want it or not, because I know she’s not eating as well, and so I want her to eat and therefore I end up eating with her (Husband, Age 80, Live-in carer).

Although family carers recognised that the increase in sweet foods had negative implications for their own health, the majority agreed that this was necessary to ensure that the person with dementia had sufficient calories as concerns over them not eating at all were greater than not eating the ‘correct’ food.

So she was, she was then, and she was eating a lot of sweet stuff. But then she's been put on some tablets and so I'm not sure it’s good or bad, but her, her desire to eat lots of sweet stuff has gone since then. But at least that was some calories. (Daughter, Age 58, Live-out carer).

The evolving palates of people with dementia has an indirect impact on the people who care for them in terms of social behaviours, food availability and concern over the wellbeing of the individual with dementia.

#### Challenges of Food-Related Tasks

Some carers reported challenges faced with shopping and preparing foods due to their family member’s condition. As dementia progresses and care needs increase, getting time to go to for the food shopping can be a challenge as the person may not be able to stay alone in the house.

I think sometimes shopping is a bit of a problem because, you know, I’m leaving him for maybe about an hour or something, but I try to do that while he’s sleeping in the morning. I mean, I do leave him on his own. And he’s been okay up to now, but I know that could change anytime, I suppose. (Wife, Age 75, Live-in carer).

This can mean the shopping trip is rushed and stressful while worrying about the person with dementia at home. This could result in forgetting items and opting for the convenient option over the healthy option when deciding what to buy. Participants also spoke about the challenges related to going shopping with the person they are caring for, resulting in the focus being on making sure their family member is well and safe and not being able to focus on the task of shopping.

If I would do the shopping myself, it would take me 10 minutes. But when you’ve got to get mum in and out of the car and then we shuffle around and then every time you speak to her….They can’t walk and talk at the same time quite often. So, yeah. God, it takes ages. Absolutely ages. And when she gets in there, it’s completely overwhelming for her. But she wants to keep going. I’ll come away from the shops and I haven’t bought anything for myself. So not only do I not buy it then I’ve not got energy left to cook it. (Daughter, Age 54, Live-out carer)

However, some family carers did discuss how they have adapted their shopping habits to their situation to remove the stress of going to the shops, by having shopping delivered or using click and collect services.

My bugbear is doing the weekly shop. With [person with dementia] it’s a nightmare. So, because he will forget what you’ve picked. Well, I just don’t want to go there. So, my … What I have learned thanks to COVID is I do click and collect at [supermarket]. So, I do everything online. (Wife, Age 62, Live-in carer

When it came to preparing food, family carers reported having to adapt their approach as well. There were many reasons for this, including to meet the abilities of the person with dementia so they can maintain independence in feeding themselves.

… becoming less able to cut up his food. So, I tend to kind of make things that stick together, if you know what I mean? You know, like mince and potatoes and that kind of stuff that he can, you know, maybe manage with a spork or something. (Wife, Age 75, Live-in carer).

Further, the changes in taste and abilities to communicate can have an impact on how the family carer prepares food.

… she can make very pointed and very personal comments to me about stuff I cook sometimes, you overcook this, when she’s never said anything before. So, I’ve had to change how I cook things more or less. (Husband, Age 58, Live-in carer).

For some family carers, they have had to learn how to cook and shop for the household when it had previously been the role of the person living with dementia. This meant that food choices, and common family meals might not always be available.

I never did any cooking, ever. It was never something which my mother expected us to do, and it was never something which my wife expected me to do. So, I’m afraid that this whole new idea of managing the household is a new experience. (Husband, Age 80, Live-in carer).

In one situation, this new role in the house allowed the person to explore what a healthy diet meant and implement changes in their diet to positively impact both of their health.

… because I wasn’t in charge of the cooking and the shopping. I paid very little interest to it. So, so, so long as food was tasty. That was the only you know, that was the only sort of measure I applied to the food that I ate. Is it tasty or is it not tasty? Whereas now I apply an additional measure, which is it actually doing me and my wife good? (Husband, Age 69, Live-in carer).

A dementia diagnosis can impact the way families shop for, prepare and consume food. This impacts the nutrition of the family members in caring roles as well as the person living with dementia. Although there are some adaptations that can be made, it seems to bring additional stress and worry to the lives of people caring for those with dementia.

## Discussion

This study is the first to explore the factors that influence the dietary behaviours of carers of persons with dementia in conjunction with self-reported dietary intake. The results indicated that family carers of persons with dementia fail to meet current dietary recommendations for saturated fat, sugar, fibre, and fruit and vegetables but had a moderate diet quality. They were food secure, however, the findings from the semi-structured interviews demonstrated that caring for a person with dementia could influence their own dietary behaviours, beyond financial reasons.

The findings of the current study are in line with existing evidence that being a carer can result in negligence of one’s own health-promoting (including dietary) behaviours (Bailey et al., 2019; Gallant & Connell, 1998). In contrast, Uemura and Hirakawa (2020) found that some family carers of persons with dementia placed a high priority on healthy food choices, including limiting sugar intake, to remain in good health. While this be related to cultural differences between studies, a limitation of that study is it reported self-perceived dietary habits. Here we report that carers’ diets were high in saturated fats and free sugars and low in fruit and vegetable and fibre intake which was corroborated by their overall diet quality demonstrating a higher intake of red meat, commercial sweets and pastries, and a lower intake of fruit and pulses, as shown in Figure 2, when compared to the healthy Mediterranean dietary pattern. This was despite those interviewed having clear knowledge of what constitutes a healthy diet with most referencing the “importance of fruit and vegetables”, indicating a knowledge-behaviour gap, with the qualitative interviews suggesting that other factors such as historical and current attitudes to nutrition and motivations to eating healthy potentially playing a more important role in their dietary behaviours in line with the findings of others (Bloom et al., 2017; Eglseer et al., 2025). Therefore, providing nutritional information to family carers of persons with dementia is unlikely to be an effective way to initiate behaviour change and instead any education may want to place emphasis on the importance of diet for providing effective care and staying healthy and support that addresses both the demands of caregiving and the shifting nutritional needs of their loved ones.

It is important to acknowledge that this does not necessarily reflect poorer dietary behaviours in carers compared to the general UK population, in which only ∼13% and ∼28% of adults meet free sugar and 5-A-Day recommendations, respectively (SACN, 2021). Moreover, their MEDAS diet quality score was comparable to large-scale study in the UK population (Shannon et al., 2023). However, the findings of the qualitative interviews highlight some additional barriers to healthy eating directly related to their role as a carer of a person with dementia. For example, changes in eating behaviours, diet, and appetite frequently occur in dementia, with cravings and preference for sweet foods being a prominent and commonly reported feature (Fostinelli et al., 2020; Ikeda et al., 2002). Family carers have previously reported feeling guilt regarding the nutritional value of the food provided (such as discretionary and convenience foods), whilst still wanting to ensure the person with dementia is eating and enjoying food (Mole et al., 2021). In the current study this meant carers were more likely to buy and have access to these foods (e.g., cakes and biscuits), which were also offered in support groups creating additional temptations, whereby ease and access to foods are linked to their intake (Blechert et al., 2016; Maas et al., 2012). Moreover, carers reported omitting certain foods to accommodate the person with dementia's preferences, including foods that they knew were important for their overall health such as fish and vegetables, affecting their overall dietary quality.

Additionally, carers reported challenges with shopping, preparing food, and changing roles consistent with the findings of others (Hogan et al., 2004; Papachristou et al., 2013). However, the current study adds to our understanding of how these factors can specifically impact on the carers’ own dietary behaviours, for example by limiting their time and energy to devote to mealtime tasks and changing the foods they eat to maintain their loved one’s independence. On the other hand, this change in roles was viewed as a positive for one carer, giving them a chance to develop an interest in nutrition and shape improved dietary behaviours. The qualitative interviews also indicated that the impact of this was dependent on the stage of dementia and the family carers’ living and employment status. Co-residing with the person with dementia appeared to be associated with more significant dietary compromises in our qualitative data. Additional demographic factors may also have influenced the dietary behaviours observed. For example, older carers may face age-related changes in appetite, energy needs, or comorbidities that affect eating patterns (Roberts & Rosenberg, 2006). Similarly, socioeconomic status and education influence dietary behaviours, with lower levels often linked to poorer diet quality due to limited resources, food access, and nutrition knowledge (Darmon & Drewnowski, 2008). Therefore, demographic variables, such as age, education level, employment status, and living situation, interact with caregiving demands to shape dietary decisions in complex ways. However, due to its exploratory nature this study had a small sample size and was underpowered to explore these factors in detail. Moreover, as shown in Table 1 most of the participants were well educated and from less deprived areas, limiting the applicability of our findings to more diverse or underserved populations. Future research should assess dietary behaviours and the influence of caring in a larger and more diverse sample of family carers of persons with dementia.

Another subtheme ‘emotional eating’ centred around family carers turning to food and alcohol for comfort. Tangelo and colleagues (2018) assessed family carers of persons with dementia unmet needs and also found that they reported eating unhealthy foods to treat or comfort themselves when they felt stressed. Moreover, previous studies have shown that family cares may be more likely to engage in harmful alcohol consumption (Bailey et al., 2019; Denham et al., 2020). Given that caring for someone with dementia can be a particularly demanding and distressing role for the family carer (Feast et al., 2016), potential interventions should address the cognitive and emotional aspects of caregiving stress to support healthier dietary behaviours. For example, enhancing self-efficacy through behavioural interventions has been shown to significantly reduce emotional eating behaviours in family carers (MacDougall & Steffen, 2017).

### Practical Implications

The findings of this study highlight the need for practical, flexible, and personalised approaches to support the nutritional well-being of family carers of persons with dementia that address time constraints, emotional stress, and role changes experienced by carers. Evidence suggests that tailored interventions, such as digital health interventions can lead to meaningful improvements in emotional self-regulation strategies in carers (Zhai et al., 2023). Additional strategies may include grocery and/or meal delivery services (Zhu & An, 2013), embedded nutrition support for carers within dementia care pathway and community services (Gitlin et al., 2006). Additionally, healthy food provisions and peer-support networks in carer support services could promote healthier dietary patterns (Moore et al., 2019). These approaches should be adaptable to diverse caregiving situations and consider demographic factors and intervention accessibility.

### Strengths and Limitations

Despite this study benefitting from a mixed-methods design providing an in depth understanding of the dietary behaviours of family carers of persons with dementia, the current study has limitations that warrant discussion. Firstly, the sample size (n = 30 for the survey; n = 10 for interviews), while appropriate for an exploratory, mixed-methods design, limits the statistical power and may affect the generalisability of findings. Moreover, recruitment through social media and carer organisations may have resulted in a sample of more health-conscious or digitally connected individuals, potentially introducing selection bias. Furthermore, despite using a validated 24-h dietary recall, assessment of dietary intake is subject to misreporting (Poslusna et al., 2009), which should be taken into consideration when interpreting the quantitative findings. The macro-level convergent analysis approach means that individual-level qualitative observations were not paired with quantitative findings, limiting the ability to compare MEDAS scores with demographic characteristics. Finally, the cross-sectional design of the study limits our understanding of how dietary behaviours evolve with changes in caregiving demands or dementia progression, warranting additional longitudinal studies.

### Conclusion

In summary, this study highlights the complex interplay between caregiving responsibilities and the dietary behaviours of family carers of persons with dementia. Despite moderate diet quality comparable to the general UK population, carers frequently failed to meet recommendations for saturated fat, sugar, fibre, and fruit and vegetable intake, reflecting a knowledge-behaviour gap potentially influenced by the stress and demands of providing care. Key barriers included limited time and energy, adjustments to fit persons with dementia's needs, and the accessibility of discretionary foods, which often led to compromises in dietary quality. Emotional eating was identified as a significant coping mechanism, emphasising the need for interventions that address the cognitive and emotional stressors of caregiving. To better support family carers of persons with dementia, future research should explore how dietary behaviours evolve with caregiving demands, investigate the demographic and psychological factors influencing emotional eating, and develop personalised dietary interventions that address practical, cognitive, and emotional challenges to improve carer well-being.

## Supplemental Material

Supplemental Material - Having Their Cake and Eating it Too: An Exploratory Mixed-Methods Study on the Dietary Behaviours of Family Carers of Persons With Dementia Supplemental Material for Having Their Cake and Eating it Too: An Exploratory Mixed-Methods Study on the Dietary Behaviours of Family Carers of Persons With Dementia by Michael Smith, Natalie Dickinson, Nicholas Sculthorpe, Louise Ritchie, and Rachel Kimble in Dementia

## Data Availability

Data will be made available upon reasonable request.*

## References

[bibr1-14713012251355832] Afshin A. Sur P. J. Fay K. A. Cornaby L. Ferrara G. Salama J. S. Abebe Z. (2019). Health effects of dietary risks in 195 countries, 1990–2017: A systematic analysis for the global burden of disease study 2017. The Lancet, 393(10184), 1958–1972. 10.1016/S0140-6736(19)30041-8 PMC6899507 30954305

[bibr2-14713012251355832] Bailey J. M. Regan T. W. Bartlem K. M. Wiggers J. H. Wye P. M. Bowman J. A. (2019). A survey of the prevalence of modifiable health risk behaviours among carers of people with a mental illness. BMC Public Health, 19(1240), 1–10. 10.1186/s12889-019-7577-4 31500598 PMC6734289

[bibr3-14713012251355832] Blechert J. Klackl J. Miedl S. F. Wilhelm F. H. (2016). To eat or not to eat: Effects of food availability on reward system activity during food picture viewing. Appetite, 99(April 1st 2016), 254–261. 10.1016/j.appet.2016.01.006 26796027

[bibr4-14713012251355832] Bloom I. Lawrence W. Barker M. Baird J. Dennison E. SayerRobinson A. A. S. (2017). What influences diet quality in older people? A qualitative study among community-dwelling older adults from the hertfordshire cohort study, UK. Public Health Nutrition, 20(15), 2685–2693. 10.1017/S1368980017001203 28724471 PMC5612401

[bibr5-14713012251355832] Braun V. Clarke V. (2006). Using thematic analysis in psychology. Qualitative Research in Psychology, 3(2), 77–101. 10.1191/1478088706qp063oa

[bibr6-14713012251355832] Braun V. Clarke V. Hayfield N. Davey L. Jenkinson E. (2023). Doing reflexive thematic analysis. In Supporting research in counselling and psychotherapy: Qualitative, quantitative, and mixed methods research (pp. 19–38). Springer.

[bibr7-14713012251355832] Carpenter C. A. Miller M. C. Sui X. West D. S. (2020). Weight status and sedentary behavior of alzheimer's disease caregivers. American Journal of Health Behavior, 44(1), 3–12. 10.5993/AJHB.44.1.1 31783927

[bibr8-14713012251355832] Cipriani G. Carlesi C. Lucetti C. Danti S. Nuti A. (2016). Eating behaviors and dietary changes in patients with dementia. American Journal of Alzheimer’s Disease and Other Dementias, 31(8), 706–716. 10.1177/1533317516673155 PMC10852764 27756815

[bibr9-14713012251355832] Creswell J. W. Clark V. L. P. (2017). Designing and conducting mixed methods: Sage Publications.

[bibr10-14713012251355832] Darmon N. Drewnowski A. (2008). Does social class predict diet quality?1. The American Journal of Clinical Nutrition, 87(5), 1107–1117. 10.1093/ajcn/87.5.1107 18469226

[bibr11-14713012251355832] Denham A. M. J. Wynne O. Baker A. L. Spratt N. J. Turner A. Magin P. Palazzi K. Bonevski B. (2020). An online cross-sectional survey of the health risk behaviours among informal caregivers. Health Promotion Journal of Australia: Official Journal of Australian Association of Health Promotion Professionals, 31(3), 423–435. 10.1002/hpja.296 31529552

[bibr53-14713012251355832] Eglseer D Bauer S Farzer E Reitbauer M Lampersberger L (2025). Barriers, facilitators, and needs for supporting healthy diets in community-dwelling older adults and their informal caregivers: A qualitative study. Appetite, 214(2025), 108184. 10.1016/j.appet.2025.108184 40484238

[bibr13-14713012251355832] Feast A. Orrell M. Charlesworth G. Melunsky N. Poland F. Moniz-Cook E. (2016). Behavioural and psychological symptoms in dementia and the challenges for family carers: Systematic review. The British Journal of Psychiatry: The Journal of Mental Science, 208(5), 429–434. 10.1192/bjp.bp.114.153684 26989095 PMC4853642

[bibr14-14713012251355832] Foster E. Lee C. Imamura F. Hollidge S. E. Westgate K. L. Venables M. C. Brage S. Rowland M. K. Osadchiy T. Bradley J. C. Simpson E. L. Adamson A. J. Olivier P. Wareham N. Forouhi N. G. (2019). Validity and reliability of an online self-report 24-h dietary recall method (Intake24): A doubly labelled water study and repeated-measures analysis. Journal of Nutrition Sciences, 8(2019), Article e29. 10.1017/jns.2019.20 PMC6722486 31501691

[bibr15-14713012251355832] Fostinelli S. De Amicis R. Leone A. Giustizieri V. Binetti G. Bertoli S. Battezzati A. Cappa S. F. (2020). Eating behavior in aging and dementia: The need for a comprehensive assessment. Frontiers in Nutrition, 7(e2022), Article 604488. 10.3389/fnut.2020.604488 PMC7772186 33392240

[bibr16-14713012251355832] Gallant M. P. Connell C. M. (1998). The stress process among dementia spouse caregivers:are caregivers at risk for negative health behavior change? Research on Aging, 20(3), 267–297. 10.1177/0164027598203001

[bibr17-14713012251355832] Gitlin L. N. Reever K. Dennis M. P. Mathieu E. Hauck W. W. (2006). Enhancing quality of life of families who use adult day services: Short- and long-term effects of the adult day services plus program. The Gerontologist, 46(5), 630–639. 10.1093/geront/46.5.630 17050754

[bibr18-14713012251355832] Guasch-Ferré M. Willett W. (2021). The mediterranean diet and health: A comprehensive overview. Journal of Internal Medicine, 290(3), 549–566. 10.1111/joim.13333 34423871

[bibr19-14713012251355832] Hogan V. M. Lisy E. D. Savannah R. L. Henry L. Kuo F. Fisher G. S. (2004). Role change experienced by family caregivers of adults with Alzheimer’s disease: Implications for occupational therapy. Physical & Occupational Therapy in Geriatrics, 22(1), 21–43. 10.1080/J148v22n01_02

[bibr20-14713012251355832] Hu F. B. (2002). Dietary pattern analysis: A new direction in nutritional epidemiology. Current Opinion in Lipidology, 13(1), 3–9. 10.1097/00041433-200202000-00002 11790957

[bibr21-14713012251355832] Ikeda M. Brown J. Holland A. J. Fukuhara R. Hodges J. (2002). Changes in appetite, food preference, and eating habits in frontotemporal dementia and Alzheimer’s disease. Journal of Neurology, Neurosurgery & Psychiatry, 73(4), 371–376. 10.1136/jnnp.73.4.371 12235302 PMC1738075

[bibr22-14713012251355832] Jaron D. Galal O. (2009). Food security and population health and well being. Asia Pacific Journal of Clinical Nutrition, 18(4), 684–687. 19965366

[bibr23-14713012251355832] Johanson G. A. Brooks G. P. (2010). Initial scale development: Sample size for pilot studies. Educational and Psychological Measurement, 70(3), 394–400. 10.1177/0013164409355692

[bibr24-14713012251355832] Kasper J. D. Freedman V. A. Spillman B. C. Wolff J. L. (2015). The disproportionate impact of dementia on family and unpaid caregiving to older adults. Health Affairs, 34(10), 1642–1649. 10.1377/hlthaff.2015.0536 26438739 PMC4635557

[bibr25-14713012251355832] Laraia B. A. (2013). Food insecurity and chronic disease. Advances in Nutrition, 4(2), 203–212. 10.3945/an.112.003277 23493536 PMC3649100

[bibr26-14713012251355832] Lindeza P. Rodrigues M. Costa J. Guerreiro M. Rosa M. M. (2024). Impact of dementia on informal care: A systematic review of family caregivers’ perceptions. BMJ Supportive & Palliative Care, 14(e1), e38–e49. 10.1136/bmjspcare-2020-002242 33055092

[bibr27-14713012251355832] Maas J. de Ridder D. T. D. de Vet E. de Wit J. B. F. (2012). Do distant foods decrease intake? The effect of food accessibility on consumption. Psychology and Health, 27(sup2), 59–73. 10.1080/08870446.2011.565341 21678172

[bibr28-14713012251355832] MacDougall M. Steffen A. (2017). Self-efficacy for controlling upsetting thoughts and emotional eating in family caregivers. Aging & Mental Health, 21(10), 1058–1064. 10.1080/13607863.2016.1196335 27323869

[bibr29-14713012251355832] Martínez-González M. A. García-Arellano A. Toledo E. Salas-Salvado J. Buil-Cosiales P. Corella D. Covas M. I. Schröder H. Arós F. Gómez-Gracia E. Fiol M. Ruiz-Gutiérrez V. Lapetra J. Lamuela-Raventos R. M. Serra-Majem L. Pintó X. Muñoz M. A. Wärnberg J. Estruch R. (2012). A 14-item mediterranean diet assessment tool and obesity indexes among high-risk subjects: The PREDIMED trial. PLoS One, 7(8), e43134. 10.1371/journal.pone.0043134 22905215 PMC3419206

[bibr30-14713012251355832] Miller V. Webb P. Micha R. Mozaffarian D. Global Dietary Database . (2020). Defining diet quality: A synthesis of dietary quality metrics and their validity for the double burden of malnutrition. The Lancet Planetary Health, 4(8), e352–e370. 10.1016/S2542-5196(20)30162-5 32800153 PMC7435701

[bibr31-14713012251355832] Mole L. Kent B. Abbott R. Hickson M. (2021). Family carers’ experiences of nutritional care for people living with dementia at home: An interpretative phenomenological analysis. Dementia, 20(1), 231–246. 10.1177/1471301219872032 31488020 PMC7940801

[bibr32-14713012251355832] Moore S. E. McMullan M. McEvoy C. T. McKinley M. C. Woodside J. V. (2019). The effectiveness of peer-supported interventions for encouraging dietary behaviour change in adults: A systematic review. Public Health Nutrition, 22(4), 624–644. 10.1017/S1368980018003294 30501679 PMC6411137

[bibr33-14713012251355832] Owen L. Corfe B. (2017). The role of diet and nutrition on mental health and wellbeing. Proceedings of the Nutrition Society, 76(4), 425–426. 10.1017/S0029665117001057 28707609

[bibr34-14713012251355832] Papachristou I. Giatras N. Ussher M. (2013). Impact of dementia progression on food-related processes: A qualitative study of caregivers’ perspectives. American Journal of Alzheimer's Disease and Other Dementias, 28(6), 568–574. 10.1177/1533317513494456 PMC10852594 23813792

[bibr35-14713012251355832] Pool U. Dooris M. (2022). Prevalence of food security in the UK measured by the food insecurity experience scale. Journal of Public Health, 44(3), 634–641. 10.1093/pubmed/fdab120 33866365 PMC8083270

[bibr54-14713012251355832] Poslusna K Ruprich J de Vries JHM Jakubikova M van’t Veer P (2009). Misreporting of energy and micronutrient intake estimated by food records and 24 hour recalls, control and adjustment methods in practice. British Journal of Nutrition, 101(S”), S73–S85. 10.1017/S0007114509990602 19594967

[bibr36-14713012251355832] Richardson T. J. Lee S. J. Berg-Weger M. Grossberg G. T. (2013). Caregiver health: Health of caregivers of Alzheimer’s and other dementia patients. Current Psychiatry Reports, 15(367), 1–7. 10.1007/s11920-013-0367-2 23712718

[bibr37-14713012251355832] Roberts S. B. Rosenberg I. (2006). Nutrition and aging: Changes in the regulation of energy metabolism with aging. Physiological Reviews, 86(2), 651–667. 10.1152/physrev.00019.2005 16601270

[bibr38-14713012251355832] Rullier L. Lagarde A. Bouisson J. Bergua V. Torres M. Barberger-Gateau P. (2014). Psychosocial correlates of nutritional status of family caregivers of persons with dementia. International Psychogeriatrics, 26(1), 105–113. 10.1017/S1041610213001579 24047643

[bibr39-14713012251355832] Saarijärvi M. Bratt E. L. (2021). When face-to-face interviews are not possible: Tips and tricks for video, telephone, online chat, and email interviews in qualitative research. Oxford University Press. 10.1093/eurjcn/zvab038 PMC8135391 33893797

[bibr40-14713012251355832] SACN . (2021). SACN statement on nutrition and older adults living in the community. https://www.gov.uk/government/publications/sacn-statement-on-nutrition-and-older-adults

[bibr41-14713012251355832] Sehar U. Rawat P. Reddy A. P. Kopel J. Reddy P. H. (2022). Amyloid beta in aging and Alzheimer’s disease. International Journal of Molecular Sciences, 23(21), Article 12924. 10.3390/ijms232112924 36361714 PMC9655207

[bibr42-14713012251355832] Shannon O. M. Ranson J. M. Gregory S. Macpherson H. Milte C. Lentjes M. Mulligan A. McEvoy C. Griffiths A. Matu J. Hill T. R. Adamson A. Siervo M. Minihane A. M. Muniz-Tererra G. Ritchie C. Mathers J. C. Llewellyn D. J. Stevenson E. (2023). Mediterranean diet adherence is associated with lower dementia risk, independent of genetic predisposition: Findings from the UK biobank prospective cohort study. BMC Medicine, 21(1), 81. 10.1186/s12916-023-02772-3 36915130 PMC10012551

[bibr43-14713012251355832] Slawson D. L. Fitzgerald N. Morgan K. T. (2013). Position of the academy of nutrition and dietetics: The role of nutrition in health promotion and chronic disease prevention. Journal of the Academy of Nutrition and Dietetics, 113(7), 972–979. 10.1016/j.jand.2013.05.005 23790411

[bibr44-14713012251355832] Tatangelo G. McCabe M. Macleod A. You E. (2018). “I just don’t focus on my needs.” the unmet health needs of partner and offspring caregivers of people with dementia: A qualitative study. International Journal of Nursing Studies, 77(January 2018), 8–14. 10.1016/j.ijnurstu.2017.09.011 28982034

[bibr45-14713012251355832] Tombini M. Sicari M. Pellegrino G. Ursini F. Insarda P. Di Lazzaro V. (2016). Nutritional status of patients with Alzheimer’s disease and their caregivers. Journal of Alzheimer’s Disease, 54(4), 1619–1627. 10.3233/JAD-160261 27636839

[bibr46-14713012251355832] Tsapanou A. Zoi P. Sakka P. (2024). Sleep, diet, and exercise: How much dementia caregivers are affected? Brain Sciences, 14(8), 826. 10.3390/brainsci14080826 39199517 PMC11352422

[bibr47-14713012251355832] Uemura M. Y. Hirakawa Y. (2020). Self-perceived eating habits among family caregivers of older people with dementia: A qualitative study. Journal of Nutrition in Gerontology and Geriatrics, 39(3-4), 205–213. 10.1080/21551197.2020.1819510 32930643

[bibr48-14713012251355832] Vitaliano P. P. Russo J. Scanlan J. M. Greeno C. G. (1996). Weight changes in caregivers of Alzheimer's care recipients: Psychobehavioral predictors. Psychology and Aging, 11(1), 155–163. 10.1037//0882-7974.11.1.155 8726381

[bibr49-14713012251355832] Walker-Clarke A. Walasek L. Meyer C. (2022). Psychosocial factors influencing the eating behaviours of older adults: A systematic review. Ageing Research Reviews, 77(May 2022), Article 101597. 10.1016/j.arr.2022.101597 35219902

[bibr50-14713012251355832] Wittenberg R. Hu B. Barraza-Araiza L. Rehill A. (2019). Projections of older people with dementia and costs of dementia care in the United Kingdom, 2019–2040 (vol. 2). London School of Economics.

[bibr51-14713012251355832] Zhai S. Chu F. Tan M. Chi N. C. Ward T. Yuwen W. (2023). Digital health interventions to support family caregivers: An updated systematic review. Digital health, 9(e2023), Article 20552076231171967. 10.1177/20552076231171967 PMC10201006 37223775

[bibr52-14713012251355832] Zhu H. An R. (2013). Impact of home-delivered meal programs on diet and nutrition among older adults: A review. Nutrition and Health, 22(2), 89–103. 10.1177/0260106014537146 24916974

