# Supplementary material for: Having Their Cake and Eating it Too: An Exploratory Mixed-Methods Study on the Dietary Behaviours of Family Carers of Persons With Dementia
Source: Dementia (London). 2025 Jul 23;25(5):1019–38. doi: 10.1177/14713012251355832 (PMC13260743; doi:10.1177/14713012251355832)
Supplement: Supplemental Material - Having Their Cake and Eating it Too: An Exploratory Mixed-Methods Study on the Dietary Behaviours of Family Carers of Persons With Dementia [file sj-pdf-1-dem-10.1177_14713012251355832.pdf]

Interview Moderator Guide

Introduction*: Thank you for agreeing to participate in this interview. As you know, we are interested in understanding the relationship between caring for a person living with dementia and diet, in particular around healthy eating. We have invited you to take part in this interview because you have indicated to us you are in a caring role for your [insert relationship parent/spouse etc.] who is living with dementia. Your insights are invaluable in shedding light on this important topic. The interview will take approximately 60 minutes. Your responses will remain confidential, and the data collected will be used solely for research purposes.*

Section 1: Participant Information and caregiving experience

1. How long have you been the primary caregiver for your husband/wife/mother/father?

2. Can you describe a typical day in your role as a caregiver?

3. What are the main nutritional responsibilities and challenges you face as a caregiver?

Topics to be covered include:

1. Caregiving role of participant
2. Understanding of the impact of caregiving on diet and nutrition
3. Barriers to maintaining a healthy diet
4. Facilitators to maintaining a healthy diet
5. Advice received around diet and nutritional wellbeing
6. Views on what advice is required for carers
7. Emerging themes – anything else you'd like to discuss that you think is important

The researcher will cover each topic in a conversational manner, prompts will be used as appropriate to explore topics.

Prompts:

- How has your role as a caregiver for X influenced your own dietary habits and eating patterns?
    - *Prompt: Consider changes in types of foods, meal schedules, etc.*

- People often talk about having a 'healthy diet'. What does that mean to you? How has caregiving for X affected your understanding of dietary recommendations?
    - *Probe: Any specific areas of uncertainty?*

- Could you describe any challenges you encounter when trying to eat healthily while caregiving?
    - *Probes: Time constraints, stress impact, lack of energy, access to nutritious foods, less social interactions during meals*
    - *Follow up: How do you manage these challenges?*
    - *Follow-up: Have you noticed any patterns of using food for coping?*

- Have you identified any strategies, practices, or resources that have helped you maintain a healthy diet despite the demands of caregiving?

  - *Probes: Social support, tools, interventions*

- Based on your experiences, what advice or suggestions would you offer to other family carers facing similar challenges in maintaining a healthy diet while caring for a person with dementia?

- Is there anything else you would like to share about your caregiving journey, its impact on your dietary habits, and your knowledge of healthy eating?

Closing: *Thank you for sharing your valuable insights and experiences. Your participation will contribute to a deeper understanding of caregiving on family carers' diets and nutritional well-being. If you have any additional thoughts or questions after the interview, please don't hesitate to reach out. Your time and contribution are greatly appreciated.*
